# Effect of Surface-Immobilized States of Antimicrobial Peptides on Their Ability to Disrupt Bacterial Cell Membrane Structure

**DOI:** 10.3390/jfb15110315

**Published:** 2024-10-25

**Authors:** Tong Lou, Xueqiang Zhuang, Jiangfan Chang, Yali Gao, Xiuqin Bai

**Affiliations:** 1 School of Marine Engineering, Jimei University, Xiamen 361021, China; loutong@jmu.edu.cn (T.L.);; 2 Fujian Institute of Innovation for Marine Equipment Detection and Remanufacturing Industrial Technology, Xiamen 361021, China; 3 State Key Laboratory of Maritime Technology and Safety, Wuhan University of Technology, Wuhan 430063, China; 4 Reliability Engineering Institute, National Engineering Research Center for Water Transport Safety, Wuhan University of Technology, Wuhan 430063, China

**Keywords:** antimicrobial peptide, molecular dynamics simulations, membrane structure

## Abstract

Antimicrobial peptide (AMP) surfaces are widely used to inhibit biofilm formation and bacterial infection. However, endpoint-immobilized AMPs on surfaces are totally different from free-state AMPs due to the constraints of the surface. In this work, the interactions between AMPs and bacterial cell membranes were analyzed through coarse-grained molecular dynamics and all-atom molecular dynamics simulations. This AMP disrupted membrane structure by altering the thickness and curvature of the membrane. Furthermore, the effect of surface-immobilized states of AMPs on their ability to disrupt membrane structure was revealed. The immobilized AMPs in the freeze-N system could bind to the membrane and disrupt the membrane structure through electrostatic forces between positively charged N-terminal amino acid residues and the negatively charged membrane, while the immobilized AMPs in the freeze-C system were repelled. The results will aid in the rational design of new AMP surfaces with enhanced efficacy and stability.

## 1. Introduction

Biological peptides are essential participants in the fulfillment of various complex physiological activities of living organisms. Among them, antimicrobial peptides (AMPs) are part of the body’s first line of defense for pathogen inactivation and are widely used as an alternative to antibiotics to kill bacteria. AMPs have been immobilized on surfaces to prepare AMP surfaces to inhibit biofilm formation and bacterial infection of implants or other medical devices [1,2,3]. They have also been extended to the marine field to inhibit bacterial adhesion and biofilm formation on surfaces immersed in seawater [4,5,6].

The antimicrobial mechanism of AMPs is the focus of current research and involves interactions with substances such as cell membranes, enzymes, proteins and nucleic acids [7]. Clarifying the antimicrobial mechanism not only enables better application of AMPs, but also helps to establish a structure-function relationship, which facilitates the effective development of AMPs [8]. Among them, the interaction between AMPs and bacterial cell membranes is particularly critical, because it is the initial step of AMPs in killing bacteria. Subsequently, AMPs act on intracellular substances (nucleic acids, proteins, or enzymes) to affect a number of key cellular processes or target the cell membrane to destroy its structural integrity [9].

Currently, instruments such as circular dichroism spectrometers, solid-state nuclear magnetic resonance apparatus, and scanning electron microscopy can observe some fixed moments of AMPs interacting with membrane [10,11,12]. Additionally, labeling AMPs with a fluorophore can further aid in tracking their interaction with bacterial cell membranes, enabling the assessment of their killing mechanism [13,14]. However, it is not yet possible to visualize the complete dynamic process of cell membrane disruption at the molecular level under the current state of the art. Fortunately, molecular dynamics simulation as a high-precision “camera” can compensate for the shortcomings of the experimental method [15]. Coarse-grained molecular dynamics (CG MD) simulations with a Martini force field are commonly used to study the interaction of AMPs with membranes from the perspective of large systems and long term [16,17,18]. Gao et al. [19] used the CG MD simulations with the Martini2 force field to study the physical mechanism of natural AMP PaDBS1 and its two derivative peptides PaDBS1R1 (2n9r) and PaDBS1R6 (6cfa) acting on different phospholipid membranes. The simulation results showed that their action mechanism relied mainly on a combination of electrostatic interaction and hydrophobic interaction. The peptide membrane interaction gave rise to changes in the phospholipid membrane structure, such as its thickness, surface area, fluidity, and so on.

However, CG MD simulations are unable to obtain details from the atomic level due to accuracy issues. All-atom molecular dynamics (AA MD) simulations can analyze the interaction mechanism of AMPs with membranes from a more microscopic perspective. Strandberg et al. [20] analyzed the behavioral properties of the short cationic helical peptide BP100 on the membrane surface by AA MD simulations, and showed that the BP100 monomer could stably insert into the membrane through the interaction of the lysine side chain with the membrane, which was in agreement with the results of nuclear magnetic resonance hydrogen spectroscopy.

Current research has only focused on free-state AMPs, however, endpoint-immobilized AMPs on surfaces are totally different. They can only use the membrane as an antimicrobial target because of the inability to enter the cell. Importantly, immobilization limits their conformational changes and may result in reduced antimicrobial activity, thus, surface immobilization strategies are critical for the performance of AMP surfaces. AMPs are generally chemically immobilized to the surface to increase stability using coupling agents, including dopamine, polyethylene glycol, glycopolymers, and so on [21], and are often linked to coupling agents through the free amino or carboxyl groups at terminals [22]. There is a lack of studies on the effect of AMP-immobilized states on activity.

Here, we expose the effect of AMP-immobilized states (N-terminal or C-terminal is immobilized to the surfaces) on bacterial cell membrane structure through CG MD and AA MD simulations. (1) CG MD simulation. Taking the phosphate groups in the membranes as research objects, we analyze the effects of four different states of AMP molecules on membrane structure (the distribution, thickness, and curvature), clarify the mechanism of how AMP destroys the cell membranes, and compare the activities of AMP molecules in different states. (2) AA MD simulation. Taking a single antimicrobial peptide molecule as the research object, the main active sites of AMP were clarified by analyzing the relative positional relationships and interaction energies of different residues with the membrane. The two simulations provide new perspectives on the construction of AMP surfaces.

## 2. Theory and Methods

### 2.1. AMP Parameter

Since the discovery of insect aspergillin, human defensin, and frogspin, in the 1980s, the family of AMPs has been expanding [23]. As of Jan 2024, 3146 natural AMPs from the six life kingdoms are already included in the Antimicrobial Peptide Database (APD3, https://aps.unmc.edu/home (accessed on 10 September 2024)). Moronecidin-like peptide (sequence: FFRNLWKGAKAAFRAGHAAWRA, mol formula: C_120_H_177_N_37_O_24_, APD ID: AP03001) was derived from *Hippocampus comes* with antimicrobial properties, including *E. coli* (MIC 6.25 ug/mL), *P. aeruginosa* (MIC 50 ug/mL), *A. baumannii* (MIC 6.25 ug/mL), *S. aureus* or MRSA (MIC 3.1 ug/mL), *S. epidermidis* (MIC 1.6 ug/mL), *C. albicans* (MIC 6.2 ug/mL), and *C. tropicalis* (MIC 6.2 ug/mL) [24]. The antimicrobial mechanism of this AMP has not been studied at the molecular level. This peptide was used in the work only as a model to identify the effect of immobilized states of AMPs on their ability to disrupt bacterial cell membrane structure.

### 2.2. Membrane Parameter

Bacterial cell membranes are complex, with variations between different types of bacteria. Gram-positive bacteria have a cell membrane composed of a peptidoglycan layer and a cytoplasmic inner membrane, whereas Gram-negative bacteria feature an outer membrane in addition to the peptidoglycan layer and cytoplasmic inner membrane [25,26,27]. The interaction of AMPs with the cytoplasmic inner membrane is crucial for their function, as these peptides must penetrate the membrane to either enter the cell or disrupt the membrane structure to kill the bacteria. The cytoplasmic inner membrane mainly comprises phosphatidylethanolamine (PE), phosphatidylglycerol (PG), and cardiolipin (CL), typically in a molar ratio of 75:20:5 (PE:PG:CL) [28]. To simulate bacterial cell membranes, we used a lipid system with a 75:20:5 ratio of DOPE:DOPG:TOCL.

### 2.3. Coarse-Grained Molecular Dynamics Simulation

#### 2.3.1. Model Construction

**Figure 1:**
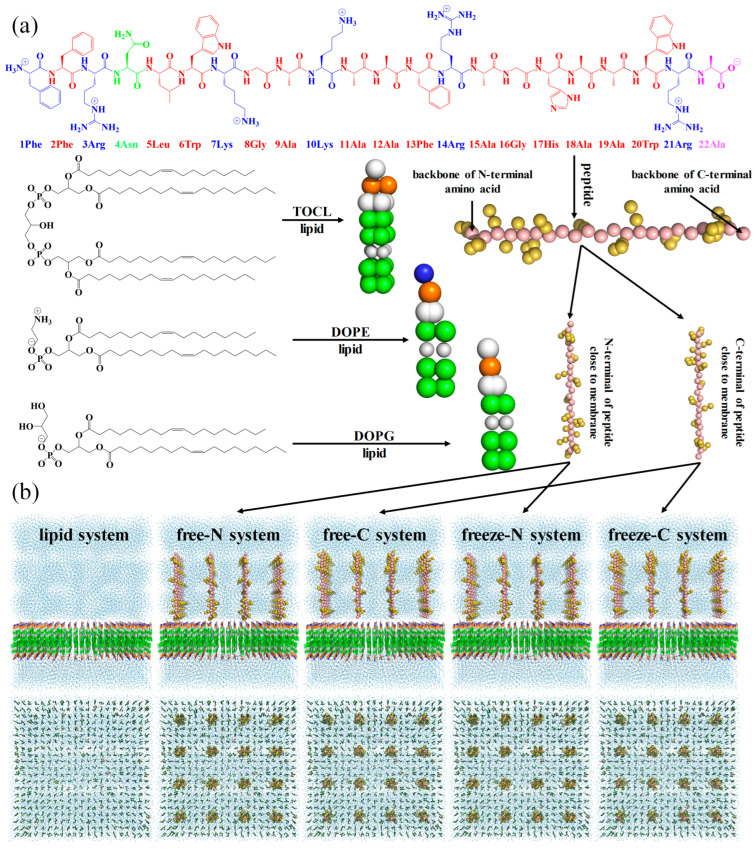
The coarse-grained molecules (**a**) and systems (**b**). For clarity, the membrane, peptide molecules, and solvent are shown as lines, sphere, and nb_spheres, respectively.

Firstly, three types of coarse-grained lipid molecules were regularly arranged in a 16 × 16 × 20 nm^3^ box using the tool Insane [29] to construct a lipid membrane. Then, for the system without peptide molecules, the charge of the system was neutralized with 0.15 M ions (Na^+^ and Cl^−^), and recorded as the lipid system. For the system with free-state peptide molecules, 16 coarse-grained peptide molecules were regularly inserted above the membrane perpendicular to the X/Y plane, the charge was also neutralized with 0.15 M ions and regarded as either the free-N system (N-terminal of peptide molecules close to the membrane) or the free-C system (C-terminal of peptide molecules close to the membrane). For the system with endpoint-immobilized peptide molecules, the backbone of all C-terminal amino acid residues (22Ala) in the free-N system was frozen to mimic the immobilized state, referred to as the freeze-N system. Similarly, the backbone of all N-terminal amino acid residues (1Phe) in the free-C system was frozen, forming the freeze-C system. The details of these configurations are shown in Table 1 and Figure 1.

#### 2.3.2. Simulation Parameter

In this work, the CG MD simulations relied on the Martini2 force field [16,17] and were executed under the GROMACS package (2019.6) with GPU acceleration. Initially, the systems were energy-optimized to eliminate the irrational spatial structure formed during model construction. Despite minimization, the systems remained energetically unfavorable due to their complexity, necessitating pre-equilibrated simulations in the NPT ensemble at 303 K with short time steps (1, 5, 10, and 40 fs). Finally, the production simulations were conducted for 10,000 ns with a time step of 0.04 ps. The temperature was coupled at 303 K using a berendsen thermostat and applied separately to lipids, peptide molecules, and solvent. A Parrinello-Rahman barostat was used for pressure coupling at 1 bar, independently for the Z-direction and X/Y-directions. 

### 2.4. All-Atom Molecular Dynamics Simulation

#### 2.4.1. Model Construction

**Figure 2:**
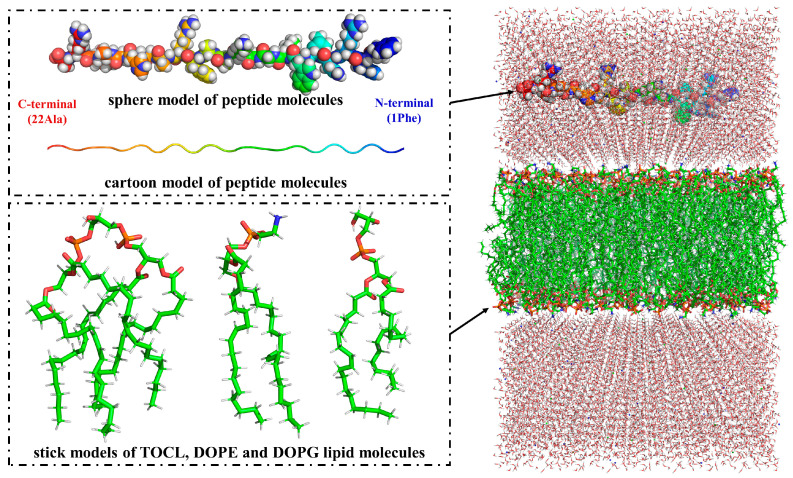
The initial structure of the AA MD system.

The lipid membrane was generated using the CHARMM-GUI tool (https://www.charmm-gui.org/) [30], consisting of 150 DOPE lipid molecules, 40 DOPG lipid molecules, and 10 TOCL lipid molecules in an 8 × 8 × 13 nm^3^ box. A peptide molecule was positioned on the top of the membrane, oriented parallel to the XY plane. To neutralize the system, 0.15 M ions were added. The initial structure of the system is illustrated in Figure 2.

#### 2.4.2. Simulation Parameter

The AA MD simulation was still conducted using the GROMACS package (2019.6) with the CHARMM36m force field [31]. Initially, the system was energy-minimized using the steepest descent minimization method. This was followed by a pre-equilibrated simulation in the NVT ensemble to regulate temperature to 303 K for 100 ps. Subsequently, another pre-equilibrated simulation was performed in the NPT ensemble for 100 ps. After these pre-equilibrated steps, the production simulation was carried out for 80 ns with a time step of 2 fs. The temperature was coupled at 303 K using a Nosé-Hoover thermostat, which was applied separately for lipids, peptide molecule and solvent. A Parrinello-Rahman barostat was used for pressure coupling at 1 bar, independently for the Z-direction and X/Y-directions.

## 3. Results and Discussion

### 3.1. Coarse-Grained Molecular Dynamics Simulation

**Figure 3:**
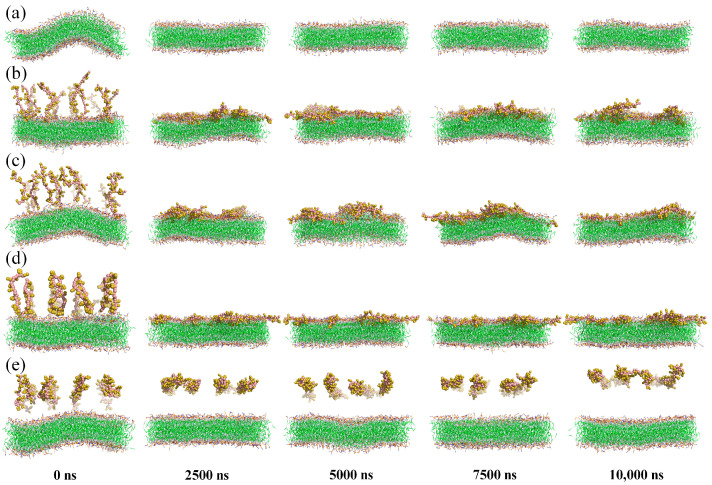
Simulation snapshots of the lipid system (**a**), free-N system (**b**), free-C system (**c**), freeze-N system (**d**) and freeze-C system (**e**) at 0, 2500, 5000, 7500, and 10,000 ns. The solvent was hidden for clarity, while membrane and peptide molecules were shown as lines and spheres.

CG MD simulations were used to comparatively analyze the effects of different states of peptide molecules on membrane structure. To visualize changes in membrane structure during the simulations, snapshots of five systems were selected every 2500 ns, as shown in Figure 3.

In the lipid system, the membrane maintained a regular parallel bilayer structure throughout the simulation, except at 0 ns (Figure 3a). This initial irregularity was due to unbalanced compressive stresses inside and outside the membrane, which resolved as the simulation progressed.

**Figure 4:**
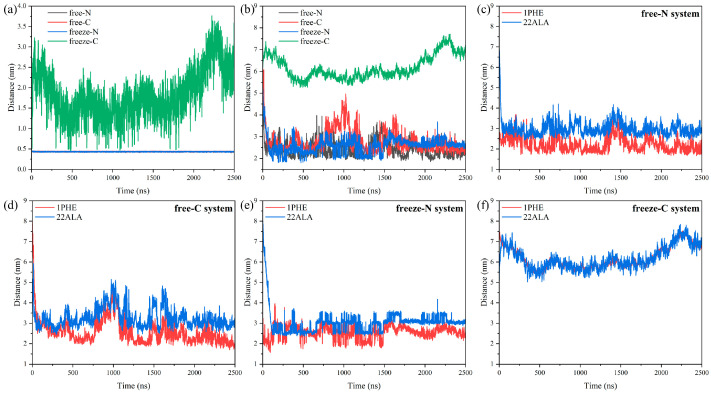
Minimum distance between peptide molecules and the membrane (**a**), distance between the center of mass of the peptide molecules and the center of mass of the membrane (**b**), distance between the center of mass of the 1Phe/22Ala and the center of mass of the membrane (**c**–**f**).

When free-state peptide molecules were introduced, they quickly bound and inserted into the membranes (Figure 3b,c). Analysis of the distances between the peptide molecules and the membrane showed that the minimum distance remained near 0.44 nm in both free-N and free-C systems (Figure 4a). In the free-N system, the distance between the centers of mass decreased directly to below 3 nm at 24 ns, whereas in the free-C system, it took 44 ns (Figure 4b). The biggest difference between the free-N and free-C systems was the orientation of the peptide molecules. It was pretty obvious that 1Phe was closer to the membrane than 22Ala after simulation in both systems (Figure 4c,d), thus, the peptide molecules in the free-C system needed to adjust conformation to bring 1Phe residues close to the membrane. The most fundamental reason for this phenomenon was that 1Phe residues with positive charges were attracted to the membrane whereas 22Ala residues with negative charges were repelled by the membrane, which also led to huge differences in the freeze-N and freeze-C systems.

In the freeze-N system, endpoint-immobilized peptide molecules induced the membrane to approach them, while in the freeze-C system, they repelled the membrane (Figure 3d,e). Simultaneously, in the freeze-N system, 1Phe residues were still closer to the membrane than 22Ala residues, similar to the free-state peptide molecules (Figure 4e). But, in the freeze-C system, the distances of the 1Phe and 22Ala residues from the membrane were nearly the same (Figure 4f), indicating that the peptide molecules interacted internally rather than with the membrane.

**Figure 5:**
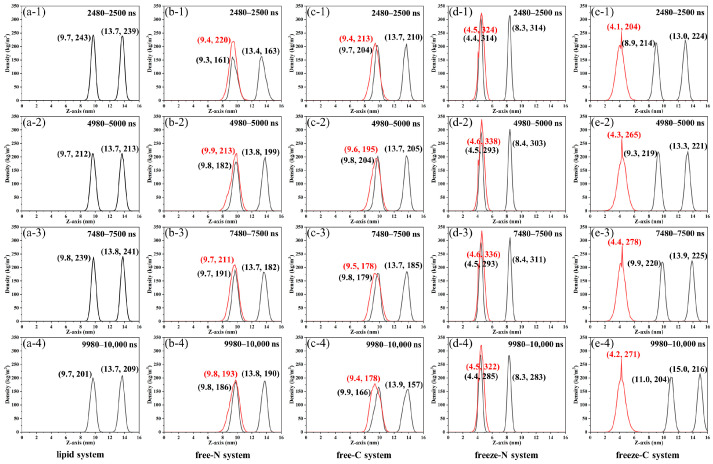
The density distributions of phosphate groups (black solid line) and peptide molecules (red solid line) along the Z-axis of the lipid system (**a**), free-N system (**b**), free-C system (**c**), freeze-N system (**d**) and freeze-C system (**e**) during 2480–2500 ns (**1**), 4980–5000 ns (**2**), 7480–7500 ns (**3**) and 9980–10,000 ns (**4**) simulations.

Phosphate groups in lipids are more reactive than the internal nonpolar carbon chains and are located on the outside of the membrane, making them useful for investigating membrane properties [4]. To further understand the relative position of the peptide molecules in relation to the membrane, the density distributions of phosphate groups and peptide molecules along the Z-axis during 2480–2500 ns, 4980–5000 ns, 7480–7500 ns, and 9980–10,000 ns simulations were analyzed (Figure 5). In the lipid system, the distribution of phosphate groups remained consistent over time (Figure 5a). In the free-N and free-C systems, the distribution range of the phosphate groups was similar to the lipid system (Figure 5b,c), indicating that the free-state peptide molecules moved toward the membrane during the simulations. According to the distribution of peptide molecules, the peptide molecules are inserted and bound to the lower layer of the membrane. Subsequently, this caused the membrane to bend and disperse phospholipid groups, which could be demonstrated by decreased peak heights in the distributions of phosphate groups.

Interestingly, phosphate groups and peptide molecules presented a completely different phenomenon in the freeze systems. In the freeze-N system, phosphate groups moved toward the peptide molecules and had the highest centralization among all systems (Figure 5d). This was because the backbone of all C-terminal amino acids in peptide molecules was frozen in the plane of Z = 4.5 nm, and the membranes were strongly attracted in the Z-direction from the endpoint-immobilized peptide molecules. The peptide molecules were similarly attracted to the membranes and concentrated at 4.5 nm or 4.6 nm. In contrast, in the freeze-C system, the membrane moved away from the peptide molecules as simulation time increased (Figure 5e). At the same time, the peptide molecules were repelled by the membrane and concentrated at 4.1–4.4 nm, which were all less than 4.5 nm. Due to less influence, the distributions of peptide molecules and phospholipid groups were more dispersed compared to the freeze-N system.

**Figure 6:**
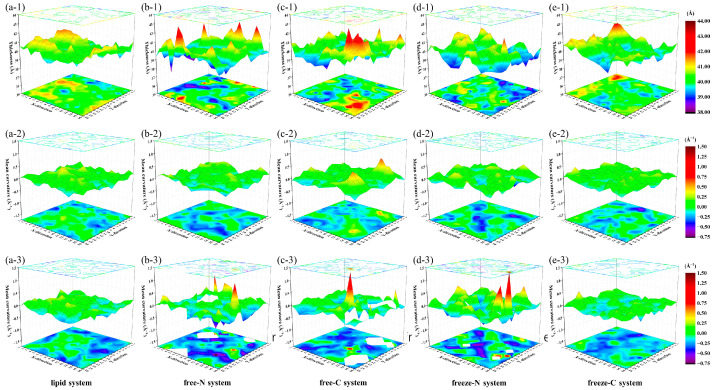
The membrane thickness (**1**), mean curvatures of upper (**2**) and lower (**3**) layers in the lipid system (**a**), free-N system (**b**), free-C system (**c**), freeze-N system (**d**) and freeze-C system (**e**) during the last 20 ns simulations.

To further extract structural information of the membrane in the X/Y plane, the X/Y plane was evenly divided into a 16 × 16 grid. The coordinates of the phosphate groups during the last 20 ns simulations in each region were extracted by the MembraneCurvature tool in the MDAnalysis library [32] to calculate membrane thickness and mean curvatures of upper and lower layers (Figure 6).

In the lipid system, the average membrane thickness was 40.3 ± 0.6 Å, the difference in membrane thickness between the region of maximum and minimum was 2.8 Å, and the curvatures of the most curved region in the upper and lower layers were 0.41 Å^−1^ and 0.34 Å^−1^, respectively. In the free-N and free-C systems, although average membrane thicknesses remained basically unchanged (39.9 ± 0.8 Å and 40.1 ± 0.8 Å), the difference in membrane thickness between the region of maximum and minimum increased to 5.0 Å and 4.8 Å, which exceeded 12% of the average membrane thickness and potentially caused fatal membrane damage [33]. The curvatures of the most curved region in the lower layers were 0.98 Å^−1^ in the free-N system and 1.36 Å^−1^ in the free-C system. Even due to the insertion of peptide molecules, curvatures could not be calculated in white regions. It has been reported that excessive curvature of the membrane could also cause membrane rupture [34,35]. Our simulations did not show obvious holes or ruptures in the membrane, so we hypothesized that the antimicrobial mechanism of this peptide cause the rupture of the membrane by excessive thickness difference and excessive bending of the local membrane.

As the peptide molecules were frozen in the freeze-N and freeze-C systems, average membrane thickness remained stable (39.8 ± 0.7 Å and 40.2 ± 0.6 Å), and the difference in membrane thickness between the region of maximum and minimum were smaller (3.7 Å and 3.2 Å) than in the free-N and free-C systems. The curvature of the most curved region in the lower layer was 1.50 Å^−1^ in the freeze-N system, compared with 0.48 Å^−1^ in the freeze-C system. This suggested that the endpoint-immobilized peptide molecules in the freeze-N system could also cause membrane rupture by excessive bending of the membrane over the simulation time period, whereas the peptide molecules in the freeze-C system did not exhibit the same properties.

The above simulations showed that immobilization of peptides on the surfaces would affect their activity and that different immobilization strategies would produce different effects (retention of activity or loss of activity). Therefore, when peptides need to be immobilized on surfaces to achieve different functions, it is necessary to identify their active groups, and then choose appropriate grafting strategies to maximize the retention of activity by exposing active groups to bacteria.

### 3.2. All-Atom Molecular Dynamics Simulation

**Figure 7:**
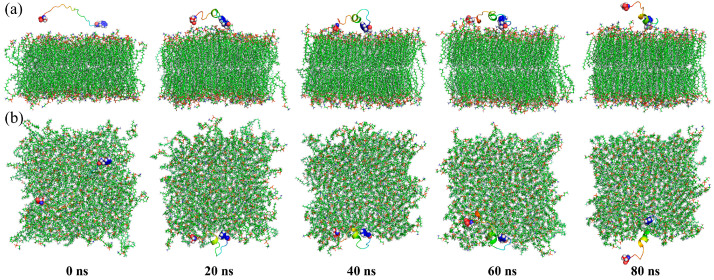
AA MD simulation snapshots of the front (**a**) and top (**b**) views at 0, 20, 40, 60, and 80 ns. For clarity, the solvent is hidden, the membrane is shown as lines, and the peptide molecule is shown as a cartoon, except for the N-terminal and C-terminal amino acids (spheres), colored in rainbow.

**Figure 8:**
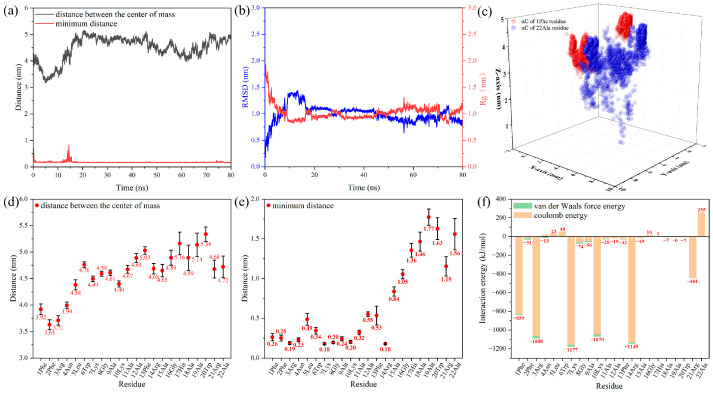
Distance between the peptide molecule and membrane (**a**), RMSD and Rg of the peptide molecule (**b**), and trajectory of αC in 1Phe and 22Ala residues (**c**) during whole simulation, distances (**d**,**e**) and interaction energy (**f**) between residues and the membrane during the last 10 ns simulation.

To further explain the differences between the five systems in the CG MD simulations, the AA MD simulation was used to study the dynamics process of an individual antimicrobial peptide molecule from contact with the membrane to the binding. Snapshots were selected every 20 ns to visualize the changes in the peptide molecule during the simulation in Figure 7. The peptide molecule was rapidly attracted to the membrane, and the minimum distance between them remained 0.17 nm after 20 ns (Figure 8a). However, due to the movement of the 22Ala residue as it was repelled by the membrane, the distance between the centers of mass fluctuated within a small range, and the root-mean-square deviation (RMSD) and radius of gyration (Rg) showed the same phenomenon (Figure 8b). The trajectory of αC in the 1Phe and 22Ala residues further suggested that the movement of the 1Phe residue was more concentrated because of the strong attraction from the membrane (Figure 8c). The last 10 ns of the trajectory was further used to analyze the properties of the stabilized system. The positional distribution of each residue was further evaluated by analyzing its distance from the membrane (Figure 8d,e). It was clear that the 1Phe residue was immobilized near the membrane while the 22Ala residue was in solution. It was evident that residues close to the N-terminal were closer to the membrane and had a smaller range of motion, which was due to binding by the membrane.

This difference was mainly due to the interaction energies between the different residues and the membrane, which could be calculated by Equation (1).

*E* = *E*_residue-membrane_ − (*E*_residue_ + *E*_membrane_) (1)

Here, *E*_residue-membrane_ represents the total energy of the residue and membrane, and *E*_residue_ and *E*_membrane_ represent the energy of the residue and membrane, respectively. It is noted that the negative value of *E* is attractive interaction energy, which is energetically conducive to residue binding membrane, and the smaller value of *E* represents the stronger bond between the residue and membrane. In contrast, the positive value of *E* is repulsive interaction energy. The results indicated that the attractive interaction energy between the peptide molecule and the membrane was −5714 kJ/mol, including the electrostatic interaction energy (−5530 kJ/mol) and van der Waals force energy (−184 kJ/mol). The electrostatic interaction energy was mainly composed of attractive interaction energy supplied by positively charged residues (1Phe, 3Arg, 7Lys, 10Lys, 14Arg, 21Arg) and repulsive interaction energy supplied by a negatively charged residue (22Ala). The three residues at the N-terminal provided 34% of the electrostatic interaction energy, which ensured stable binding of the N-terminal of the peptide molecule on the membrane. At the same time, the residues at the N-terminal were closer to the membrane, which could provide greater van der Waals force attractive energy.

The above simulation results showed that the positively charged residues near the N-terminal were bound to the membrane by electrostatic interactions, and the negatively charged residue at the C-terminal was repulsed by the membrane. This allowed the residues at the N-terminal to be close to the membrane, further enhancing the van der Waals interaction energy with the membrane. Thus, when the N-terminal of the peptide molecules was near the membrane in the freeze-N system during CG MD simulations, the membrane approached the endpoint-immobilized peptide molecules, and when the C-terminal of the peptide molecules was near the membrane in the freeze-C system, the membrane was repelled by the endpoint-immobilized peptide molecule.

## 4. Conclusions

This study explored the interactions between antimicrobial peptides and bacterial cell membranes through CG MD and AA MD simulations. In the CG MD simulations, free-state peptide molecules rapidly bound to the membrane and caused significant thickness and bending variations. The difference in membrane thickness between the region of maximum and minimum increased to 5.0 Å and 4.8 Å in the free-N and free-C systems, respectively. The curvatures of the most curved region were 0.98 Å^−1^ in the free-N system and 1.36 Å^−1^ in the free-C system. These could cause the membrane to rupture. Immobilized peptides in the freeze-N system exhibited similar properties, whereas those in the freeze-C system repelled the membrane. The reason for the difference was explained in the AA MD simulation. Positively charged N-terminal amino acid residues were attracted to the negatively charged membrane, while negatively charged C-terminal residues were repelled. This behavior was confirmed by interaction energy calculations, where the three residues at the N-terminal provided 34% of electrostatic interaction energy, which ensured the stable binding of the N-terminal of the peptide molecule on the membrane. At the same time, the residues at the N-terminal were closer to the membrane, which could provide greater van der Waals force attractive energy. Overall, the simulations revealed that the antimicrobial activity of peptides was significantly influenced by their orientation and immobilization state. Our findings highlighted that identifying active groups and selecting appropriate grafting methods are crucial for maximizing peptide efficacy when immobilized on surfaces. Future work should focus on experimental validation of these findings and further exploration of different peptide sequences and membrane compositions to develop a comprehensive understanding of peptide membrane interactions. This knowledge will aid in the rational design of new AMP surfaces with enhanced efficacy and stability.

## Figures and Tables

**Table 1:** The amounts of each component in five systems.

Systems	DOPE	DOPG	TOCL	Peptide	Na^+^	Cl^−^	Water
lipid system	660	176	44	0	727	463	29,513
free-N system	660	176	44	16	647	463	29,810
free-C system	660	176	44	16	647	463	29,810
freeze-N system	660	176	44	16	647	463	29,810
freeze-C system	660	176	44	16	647	463	29,810

## Data Availability

The original contributions presented in the study are included in the article, further inquiries can be directed to the corresponding author.
